# Bacterium-Like Particles Displaying the Rift Valley Fever Virus Gn Head Protein Induces Efficacious Immune Responses in Immunized Mice

**DOI:** 10.3389/fmicb.2022.799942

**Published:** 2022-03-17

**Authors:** Shengnan Zhang, Feihu Yan, Dongping Liu, Entao Li, Na Feng, Shengnan Xu, Hualei Wang, Yuwei Gao, Songtao Yang, Yongkun Zhao, Xianzhu Xia

**Affiliations:** ^1^Changchun Veterinary Research Institute, Chinese Academy of Agricultural Sciences, Changchun, China; ^2^The Nanjing Unicorn Academy of Innovation, Institute Pasteur of Shanghai, Chinese Academy of Sciences, Nanjing, China; ^3^College of Veterinary Medicine, Jilin University, Changchun, China

**Keywords:** Rift Valley fever (RVF) virus, bacterial like-particles, fusion protein, neutralizing antibody, specific IgG antibodies, protein anchoring

## Abstract

Rift Valley fever virus (RVFV), a mosquito-borne zoonotic phlebovirus, causes serious disease in humans and ruminants. According to the World Health Organization, Rift Valley fever is classified as a priority disease, and as such, vaccine development is of high priority due to the lack of licensed vaccines. In this study, a bacterium-like particle vaccine (BLP), RVFV-BLPs, is constructed. A novel display system is described, which is based on non-living and non-genetically modified Gram-positive bacterial cells, designated as Gram-positive enhancer matrix (GEM). The RVFV Gn head protein was displayed on the surface of GEM by co-expression with the peptidoglycan-binding domain (protein anchor) at the C-terminus. We determined that the RVFV Gn head-PA fusion protein was successfully displayed on the GEM. Mice immunized with RVFV-BLPs produced humoral and cellular immunity. Interestingly, comparing the production of RVFV Gn head-specific IgG and its subtype by vaccinating with different antigen doses of the RVFV-BLPs determined that the RVFV-BLPs (50 μg) group showed a greater effect than the other two groups. More importantly, antibodies produced by mice immunized with RVFV-BLPs (50 μg) exhibited potent neutralizing activity against RVFV pseudovirus. RVFV-BLPs (50 μg) also could induce IFN-γ and IL-4 in immunized mice; these mice generated memory cells among the proliferating T cell population after immunization with RVFV-BLPs with effector memory T cells as the major population, which means that RVFV-BLPs is an effective vaccine to establish a long-lived population of memory T cells. The findings suggest that the novel RVFV-BLPs subunit vaccine has the potential to be considered a safe and effective candidate vaccine against RVFV infection.

## Introduction

First reported in 1931 in Kenya (Daubney et al., 1931), Rift Valley fever (RVF) is a zoonotic disease caused by the RVF virus (RVFV), which primarily affects ruminants but also has the capacity to infect and cause disease in humans. Since its emergence nearly 90 years ago, RVFV has been attributed to a number of recurrent epidemics and epizootics mostly across Africa (Clark et al., 2018). As a mosquito-borne virus, RVFV is most commonly transmitted among animals and humans *via* mosquito bites; however, humans can also become infected upon exposure to infectious blood, bodily fluids, and tissues (van Velden et al., 1977; McIntosh et al., 1980; Anyangu et al., 2010; LaBeaud et al., 2015). The severity of RVFV zoonosis as well as its ability to cause major epidemics in both ruminants and humans prompted authorities to list RVFV as a notifiable disease and a potential biological weapon (Borio et al., 2002). RVFV is one of the 10 priority pathogens on the 2018 WHO Blueprint List of Priority Diseases.

RVFV (genus *Phlebovirus*; family *Phenuiviridae*; order *Bunyavirales*) is a negative-sense, single-stranded RNA virus with a tripartite genome consisting of three segments, namely, large (L), medium (M), and small (S) (Elliott, 1997; Bouloy and Weber, 2010). The L segment encodes for the viral RNA polymerase (L), whereas the M segment encodes two glycoproteins, which are cleaved during translation by cellular proteases into glycoproteins Gn and Gc, respectively (Bouloy and Weber, 2010). Additionally, the M segment encodes two non-structural proteins, namely, NSm1 and NSm2, corresponding to 78 and 14 kDa non-structural proteins, respectively (Bouloy and Weber, 2010). The S segment encodes the nucleocapsid (N) protein as well as a third non-structural protein (NSs), which functions as a virulence factor (Struthers et al., 1984). Both the viral RNA polymerase and N proteins are necessary for viral RNA replication and transcription while the glycoproteins make up the viral envelope and are strong antigenic determinants, thus eliciting potent neutralizing antibodies (Allen et al., 2018).

Historically, vaccination is proven to be an effective strategy for the prevention of viral diseases. While three veterinary vaccines are commercially available for use in endemic countries (Barteling and Woortmeyer, 1984; Minke et al., 2004; Faburay et al., 2013; Chamchod et al., 2015), there currently remains no licensed human vaccines for preventing RVFV infection (Kortekaas, 2014). However, a number of RVF vaccine platforms are reported in recent years, including a DNA vaccine (Bhardwaj et al., 2010), equine herpesvirus type 1 (EHV-1) vector vaccine (Said et al., 2017), a DNA prime with modified vaccinia virus Ankara (MVA) boost (Lorenzo et al., 2018), Newcastle disease virus (NDV)-based vector vaccine (Kortekaas et al., 2010), Lumpy skin disease virus (LSDV) vector vaccine (Wallace et al., 2020), and virus-like particle (VLP) vaccines (Mandell et al., 2010; Li et al., 2016).

In recent years, a novel antigen delivery system consisting of non-living, non-genetically modified cell wall particles derived from the food-grade bacterium *Lactococcus lactis* MG1363 is described for a number of pathogens, including shigellosis (Heine et al., 2015), human papillomavirus (HPV) (Ribelles et al., 2013), influenza virus (H1N1) (Saluja et al., 2010), porcine circovirus type 2 (PCV2) (Qiao et al., 2019), hepatitis E virus (HEV) (Gao et al., 2015), foot-and-mouth disease virus (FMDV) (Cheng et al., 2019), severe acute respiratory syndrome coronavirus (SARS) (Lee et al., 2006), and porcine reproductive and respiratory syndrome virus (PRRSV) (Li et al., 2018). This novel antigen delivery system allows for the display of multiple antigens on the particle surface in the form of recombinant fusion proteins containing a heterogeneous antigen and a peptidoglycan protein anchor (PA). The PA is derived from the C-terminus of the lactococcal cell-wall hydrolase AcmA and comprises one or more lysine motifs (LysMs), which are recognized in more than 4,000 prokaryotic and eukaryotic proteins as carbohydrate-binding protein modules (Brinster et al., 2007; Buist et al., 2008) and can be bound to the peptidoglycan (PGN) surface of bacterium-like particles (BLPs) *via* high-affinity non-covalent binding.

Here, we describe RVFV-BLPs consisting of Gram-positive enhancer matrix (GEM) particles displaying the RVFV Gn head-PA fusion protein on their surface as a novel vaccine candidate for the prevention of RVF. The RVFV Gn head-PA fusion protein was produced using a baculovirus expression system and, through the addition of a honeybee melittin signal (HBM), was successfully secreted into the supernatant. Immunization of BALB/c mice with these RVFV-BLPs resulted in RVFV-specific humoral and cellular immune responses, supporting RVFV-BLPs as a novel vaccine candidate for RVFV.

## Materials and Methods

### Ethics Statement

All BALB/c mice were purchased from Changchun Yisi Laboratory Animal Technology Co., Ltd. (Changchun, China) and kept under specific-pathogen-free (SPF) conditions, fed standard rodent chow, and provided water *ad libitum*. All BALB/c mice were handled in compliance with the guidelines for the Welfare and Ethics of Laboratory Animals of China (GB 14925-2001), and all protocols were approved by the Animal Welfare and Ethics Committee of the Veterinary Institute at the Changchun Veterinary Research Institute (Laboratory Animal Care and Use Committee Authorization, permit number JSY-DW-2018-02).

### Bacteria and Cell Culture

*Lactococcus lactis* MG1363 was generously provided to us by Jianzhong Wang and cultured in M17 medium (Qingdao Hope Bio-Technology Co., Ltd., China) supplemented with 1% glucose (GM17) (Thermo Fisher Scientific, United States) at 30°C in standing cultures. Adherent *Spodoptera frugiperda* (Sf9) cells (Life Technologies, United States) were cultured at 27°C and maintained in SFM 900 II medium (Life Technologies, United States) supplemented with 10% heat-inactivated fetal bovine serum (FBS) and 1% penicillin-streptomycin. Huh7 cells (ATCC) were cultured at 37°C with 5% CO_2_ and maintained in Dulbecco‘s modified eagle medium (DMEM, Life Technologies, United States) supplemented with 10% FBS.

### Construction of Recombinant Bacmid

**FIGURE 1:**
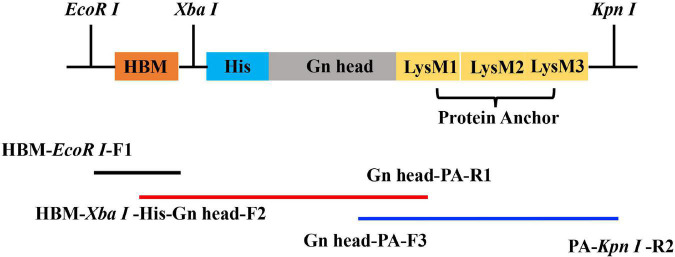
Schematic representation of the plasmid. The primers in Table 1 were used to amplify the HBM-Gn head-PA fragment according to the illustration.

The M sequence of the RVFV and the PA sequence were retrieved from the RVFV MP-12 strain (GenBank: DQ380208, 426–1,427 nt) and the *L. lactis* MG1363 strain (GenBank: U17696.1, from 820 to 1,488 nt) and were synthesized by Sangon Biotech (Shanghai, China). The Gn head domain was fused with PA and HBM (ATGAAATTCTTAGTCAACGTTGCCCT TGTTTTTATGGTCGTGTACATTTCTTACATCTATGCG) by overlap PCR using the specific primers (Table 1). The Gn head domain was designed from the fifth ATG of the M segment (426–1,427 nt), which includes a specific 54-nucleotide signal peptide (SP) sequence (Faburay et al., 2013) with an HBM signal sequence at the N-terminus and a PA sequence at C-terminus (Figure 1). The sequence was cloned into a pFast Bac 1 vector (Invitrogen-Life Technologies, United States) and confirmed by restriction enzyme digest and DNA sequencing, and then the plasmids were transformed into DH10 Bac-competent *E. coli* to generate the recombinant bacmid pFBac 1-HBM-Gn head-PA.

**TABLE 1:** Sequences of the primers used in the present study.

Primer name	Sequence (5′–3′)
HBM-*EcoRI*-F1	CG**GAATTC**ATGAAATTCTTAGTCAACGTTGCCCT TGTTTTTATGGTCGTGTACATTTCTTACA
HBM-*XbaI*-His-Gn head-F2	GTGTACATTTCTTACATCTATGCGGCCGCT**TCTA** CATCACCATCACCATCACATGACTGTGCTCCC AGCCCTG
Gn head-PA-F3	GAAGCGTGAACTGAGCGGTGCTTCTTCAGCTG
Gn head-PA-R1	CAGCTGAAGAAGCACCGCTCAGTTCACGCTTC
PA-*KpnI*-R2	GG**GGTACC**TAACTTGATACGCAGGTATTGAC CGATCAGG

*^a^Restriction enzyme sites EcoR I, Xba I, and Kpn I; are shown in bold. The HBM sequence is underlined.*

### Rescue and Identification of Recombinant Baculovirus

Rescue of the recombinant baculovirus, rpFB1-Gn head-PA, was done by transfection of Sf9 cells with the recombinant bacmid, pFBac 1-HBM-Gn head-PA, using the Cellfectin II Reagent (Life Technologies, United States). Briefly, Sf9 cells were seeded 24 h prior to transfection in a six-well plate at a density of 8 × 10^5^ cells/well to achieve a confluency of 80–90%. For transfection, 3 μg of recombinant bacmid was diluted in 100 μL Grace‘s Insect Medium (Thermo Fisher Scientific, United States) and combined with 8 μL Cellfectin II Reagent diluted in Grace‘s Insect Medium to a final volume of 210 μL. The resulting transfection mixture was vortexed briefly and incubated at room temperature for 15–30 min, after which the DNA–lipid mixture was added onto the Sf9 cells and incubated at 27°C. Supernatants containing first generation (P1) recombinant baculovirus were harvested 72 h post-transfection and passaged for three additional generations on Sf9 cells to yield fourth generation (P4) recombinant baculovirus. All passaging was done upon observation of cytopathic effects (CPE), typically within 24 h post-infection. Expression of the RVFV Gn head-PA fusion gene was confirmed by PCR (primers in Table 1) for both P3 and P4 recombinant baculovirus.

### Identification of Rift Valley Fever Virus Gn Head-Protein Anchor Fusion Protein Expressed by Recombinant Baculovirus

Expression of RVFV Gn head-PA fusion protein by the rescued recombinant baculovirus was confirmed by indirect immunofluorescence analysis (IFA). Briefly, Sf9 cells were seeded in a 96-well plate to a density of 4 × 10^4^ cells/well and infected with recombinant baculovirus at a multiplicity of infection (MOI) of 0.5 and cultured at 27°C for 48 h. Mock infected cells were included as a negative control. Following infection, the cells were fixed in 80% ice-cold acetone for 2 h at -20°C, washed with phosphate buffered saline (PBS) and incubated for 1 h at 37°C with a 1:1,000 dilution of rabbit anti-RVFV eGn serum produced in-house. Next, the cells were washed three times with PBST (0.5% Tween-20) and incubated for 1 h at 37°C with a 1:10,000 dilution of goat antirabbit IgG/FITC antibody (Bioss Antibodies, China) containing 1% Evans blue (Sigma-Aldrich, United States). The cells were then washed three times with PBST (0.5% Tween-20) and imaged using a ZEISS fluorescence microscope (Zeiss, Germany).

Both cell lysate and supernatant from recombinant baculovirus-infected Sf9 cells were subjected to Western blot analysis to confirm the expression of the RVFV Gn head-PA fusion protein. Briefly, Sf9 cells were seeded in a six-well plate at a density of 4 × 10^5^ cells/well and subsequently infected with recombinant baculovirus at an MOI of 0.5. Cell lysate and supernatants were harvested 4 days post-infection and subjected to Western blot analysis. Proteins within the cell lysate and supernatant were denatured at 95°C for 10 min and resolved on a 12% SDS-polyacrylamide gel followed by transfer onto a 0.45 μm nitrocellulose membrane (GE Healthcare Life Science, Germany) using the wet transfer method. The nitrocellulose membrane was then blocked for 2 h at room temperature with the QuickBlock Blocking Buffer (Beyotime Biotechnology, China). After blocking, the membrane was washed three times with PBST (0.5% Tween-20) and subsequently incubated overnight at 4°C with rabbit anti-RVFV eGn protein serum diluted 1:200 in QuickBlock Primary Antibody Dilution buffer (Beyotime Biotechnology, China). The following day, the membrane was washed five times (6 min/wash) with PBST (0.5% Tween-20) and then incubated for 1 h at room temperature with goat antirabbit IgG/HRP secondary antibody (Bioss Antibodies, China) diluted 1:10,000 in QuickBlock Secondary Antibody Dilution buffer (Beyotime Biotechnology, China). The nitrocellulose membrane was then washed five times (6 min/wash) in PBST (0.5% Tween-20) and Western blots imaged using the Gel Image System analysis software, version 4.2 (Tanon, China).

### Gram-Positive Enhancer Matrix Preparation

GEM particles were prepared as previously described (van Roosmalen et al., 2006). Briefly, *L. lactis* MG1363 cells were cultured in GM17, on a temperate orbital shaker at 30°C and 175 rpm for 15 h. The *L. lactis* MG1363 cells were harvested by centrifugation at 8,000 rpm for 15 min once the culture reached an OD_600_ value of 2 and the resulting cell pellet washed three times with PBS. Upon washing, the bacterial pellet was resuspended in 10% trichloroacetic acid (Sigma-Aldrich, United States) and boiled at 99°C for 30 min, effectively lysing the bacteria, degrading released proteins and DNA. The resulting lysate was washed three times with PBS and then resuspended in PBS. One unit (1U) of GEM particles was defined as approximately 2.5 × 10^9^ particles (Bosma et al., 2006). Prepared GEM particles were stored at -20°C.

### Binding and Dissociating of the Rift Valley Fever Virus Gn Head-Protein Anchor Fusion Protein to Gram-Positive Enhancer Matrix Particles

1U of GEM particles were added to the supernatant containing RVFV Gn head-PA fusion protein and slowly mixed at 37°C for 30 min. After binding, the GEM particles bound to the RVFV Gn head-PA fusion protein were collected by centrifugation at 5,000 rpm for 10 min and subsequently washed with and resuspended in PBS. RVFV Gn head-PA fusion proteins were dissociated from the GEM particles using 2% Mercaptoethanol. The mixture was then centrifuged at 10,000 rpm for 5 min and supernatants containing RVFV Gn head-PA protein collected. Samples were then analyzed by SDS-PAGE and Western blot.

### Identification of Gram-Positive Enhancer Matrix Particles Loaded With Rift Valley Fever Virus Gn Head-Protein Anchor Fusion Protein

The surface localization of RVFV Gn head-PA on GEM particles was confirmed by immunofluorescence microscopy, flow cytometry, transmission electron microscopy (TEM), and immune-electron microscopy.

For TEM, the GEM particles loaded with RVFV Gn head-PA fusion protein were prefixed with 2.5% glutaraldehyde in 0.1 M PBS (pH 7.4) for 24 h at 4°C. The GEM particles were post-fixed with 1% osmium tetroxide for 1 h at 4°C. After dehydration through a series of ethanol gradients followed by acetone solutions, the samples were embedded in Epon 812 resin mixture. Ultrathin sections (70 nm) were stained with 2% uranyl acetate in 70% ethanol and Reynold’s lead solution and examined with a JEM 1200EXII electron microscope (JEOL, Japan).

For immunofluorescence microscopy and flow cytometry, the GEM particles loaded with RVFV Gn head-PA fusion protein were fixed with 4% paraformaldehyde for 30 min at room temperature and then IFA performed as described in section “Rescue and Identification of Recombinant Baculovirus.” The GEM particles loaded with RVFV Gn head-PA fusion protein were resuspended in sterile water, GEM particles used as a negative control were treated in parallel. The samples were separated into two batches. In batch I, samples were spread on a glass slide and observed with a fluorescence microscope. In batch II, samples were analyzed by flow cytometry using a FACSAria Cell Sorter (BD Biosciences, United States).

For immune-electron microscopy, the GEM particles loaded with RVFV Gn head-PA fusion protein were absorbed on copper grids for 30 min at room temperature followed by three washes with PBS. The grids were incubated for 2 h at room temperature with rabbit anti-RVFV eGn protein serum diluted 1:200 in PBS supplemented with 2% BSA. Following incubation, the grids were washed five times with PBS and incubated for 1 h at 37°C with donkey antirabbit IgG H&L (10 nm Gold) (Abcam, United States) diluted 1:100 in 30 μL PBS. Last, grids were washed three times with PBS and examined with a JEM 1200EXII electron microscope.

### Maximum Binding Capacity of Rift Valley Fever Virus Gn Head-Protein Anchor Fusion Protein on Gram-Positive Enhancer Matrix Particles

The maximum amount of RVFV Gn head-PA fusion protein bound to 1U GEM particles was determined by SDS-PAGE. Briefly, 1U of GEM particles was combined with a range of supernatant volumes (1, 2, 3, 4, 6, 8, and 10 mL) containing the RVFV Gn head-PA fusion protein. After binding for 1 h, the complexes were washed three times with PBS. At the same time, a BSA standard was twofold serially diluted. Samples were denatured with 5 × protein loading buffer at 95°C for 10 min. Equal volumes of samples were loaded on the gel, and the samples were separated by 12% SDS-polyacrylamide gel. After Coomassie blue (Beyotime Biotechnology, China) staining for 20 min followed by 10 washes, the maximum amount of RVFV Gn head-PA fusion protein that bound to 1U GEM particles was determined based on the BSA standard using the Quantity One image analysis software.

### Vaccine Preparation and Immunization of Mice

GEM particles loaded with RVFV Gn head-PA fusion protein were defined as RVFV-bacteria like particles (RVFV-BLPs). Upon verifying that the GEM particles could bind the RVFV Gn head-PA fusion protein, we further evaluated the immunogenicity of RVFV-BLPs *in vivo* using BALB/c mice.

Six-week-old female BALB/c mice were randomly divided into four groups of 10 and each group vaccinated intramuscularly in the quadriceps with three different doses, namely, 20, 50, and 100 μg of RVFV-BLPs diluted in PBS. Mice in the control group were vaccinated with PBS. Animals in each group received a prime immunization, followed by two repeated immunizations at 2 and 4 weeks post primary immunization. Sera was harvested weekly until 4 weeks after the second repeated (third immunization).

### ELISA Measurement of Rift Valley Fever Virus Gn Head-Specific IgG Antibodies

Sera from vaccinated animals were tested for the presence of RVFV Gn head-specific IgG, IgG1, IgG2a, and IgG3 antibodies by indirect ELISA. Briefly, 96-well microtiter plates were coated overnight at 4°C with 10 μg/mL purified RVFV Gn head protein (100 μL/well) prepared in 0.05 M carbonate-bicarbonate buffer. Purified RVFV Gn head protein was produced in-house by expression in 293F cells. After coating, the plates were washed three times with PBST (0.5% Tween-20) followed by blocking for 2 h at 37°C with 5% skim milk (Solarbio, China). The plates were then incubated for 1 h at 37°C with twofold serially diluted sera prepared in 5% skim milk. Following incubation, the plates were washed three times with PBST (0.5% Tween-20) and subsequently incubated for 1 h at 37°C with 1:10,000 dilutions (100 μL/well) of either goat antimouse IgG (H&L)-HRP (Bioworld, United States), goat antimouse IgG1-HRP (Southern Biotechnology, United States), goat antimouse IgG2a-HRP (Southern Biotechnology, United States), or goat antimouse IgG3-HRP (Southern Biotechnology, United States) diluted in 5% skim milk. After washing, tetramethylbenzidine (TMB) substrate (Sigma-Aldrich, United States) was added and incubated at room temperature for 10 min in a dark room. The reaction was stopped with 50 μL/well H_2_SO_4_ (0.5 M), and the absorbance was measured at 450 nm using an automated ELISA plate reader (Thermo Fisher Scientific, United States).

### Pseudotyped Virus Neutralization Assay

An RVFV pseudotyped virus neutralization assay (PsVNA) was performed as described previously (Ma et al., 2019). Briefly, 200 pfu RVFV-pseudovirus was incubated with an equal volume of threefold serially diluted serum samples from immunized mice at 2 weeks after the third immunization in 96-well plates for 1 h at 37°C. Huh7 cells were then added to each well at a density of 3 × 10^4^ cells/well and incubated at 37°C with 5% CO_2_ for 24 h. The relative light unit (RLU) values were then measured (Nie et al., 2017), and the reduction in values was compared with those in the control wells, which were then used to determine the 50% inhibitory dilution (ID_50_) values of the sera using the Reed–Muench method.

### Lymphocyte Proliferation Assay

*In vitro* lymphocyte proliferation studies were performed with splenocytes from immunized mice. Two weeks after the third immunization, three mice from each group were randomly selected and euthanized. Harvested spleens were transferred to a tissue culture dish and mechanically minced followed by treatment in red blood cell lysing agent. The resulting splenocytes were washed with RPMI 1640 medium (Gibco, United States) containing 10% FBS. Next, the splenocytes were seeded in 96-well plates to a density of 2 × 10^3^ cells/well (100 μL/well) and stimulated for 24 h with 10 μg/mL purified RVFV Gn head protein at 37°C and 5% CO_2_. Lymphocyte proliferation was monitored according to the technical manual of the TransDetect Cell Counting kit (CCK) (TransGen Biotech, China).

### Detection of IFN-γ and IL-4 Cytokine by ELISpot Assay

ELISpot IFN-γ and IL-4 cytokine assays were performed as described previously (Zhang et al., 2019). Splenocytes were cultured in RPMI 1640 medium containing 10% FBS and stimulated with or without purified RVFV Gn head protein (10 μg/mL) for 36 h at 37°C and 5% CO_2_. Following incubation, splenocytes producing IFN-γ and IL-4 were measured using mouse enzyme-linked immunospot (ELISpot) kits (Mabtech AB, Sweden) according to the manufacturer‘s instructions. Spot-forming cells (SFCs) were enumerated using an automated ELISpot reader (AID ELISPOT reader-iSpot, Germany).

### Analysis of Splenic T Lymphocyte Subtypes

T cell subset populations and memory T lymphocytes in splenocytes were analyzed by flow cytometry. Splenocytes were seeded into six-well plates at a density of 5 × 10^6^ cells/mL and stimulated with or without purified RVFV Gn head protein (10 μg/mL) for 36 h at 37°C and 5% CO_2_. Cells were then labeled with equal volumes of 1:250 dilutions of anti-CD3-PE-Cy5, anti-CD4-FITC, anti-CD8-PE, anti-CD44-APC, and anti-CD62-PerCP-Cy5 (BD Biosciences, United States) for 30 min at 4°C. After washing, labeled cells were analyzed in a FACSAria™ Cell Sorter (BD Biosciences, United States).

## Results

### Expression of the Rift Valley Fever Virus Gn Head-Protein Anchor Fusion Protein

**FIGURE 2:**
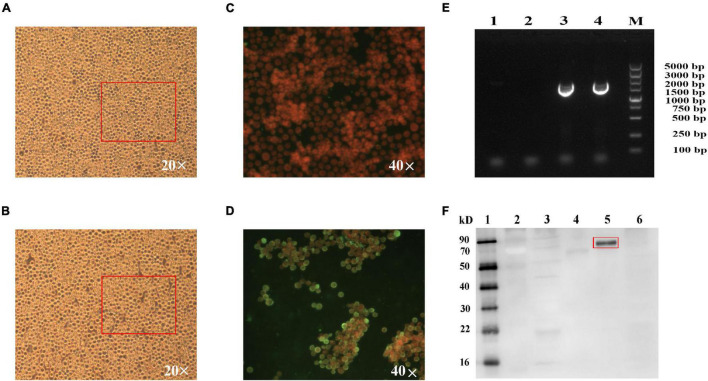
Identification of the fusion protein. Normal Sf9 cells **(A,C)**, Sf9 cells infected with 3% recombinant baculovirus for 24 h, cells showed CPE of big and round **(B)** and were fixed, permeabilized, and stained with rabbit anti-RVFV eGn serum produced in-house and fusion protein is labeled by goat antirabbit IgG/FITC **(D)**. Identification of the stability of recombinant baculovirus by PCR **(E)**, lane 1 is Sf9 cells, lane 2 is control baculovirus, and lanes 3 and 4 are the third- and fourth-generation recombinant baculovirus, RVFV Gnhead-PA is 1776 bp. The fusion proteins secreted into the cell supernatant detected by WB **(F)**, lanes 1 and 2 are a protein marker, lane 3 is Sf9 cells, lane 4 is a mixture of Sf9 cells infected with control baculovirus, the supernatant (lane 5) and cell lysis fragment (lane 6) of the Sf9 cells infected with the recombinant baculovirus. RVFV Gn head-PA is 70 kD.

The recombinant baculovirus, rpFB1-Gn head-PA, was successfully rescued by transfection of Sf9 cells with the recombinant bacmid pFBac 1-HBM-Gn head-PA. Sf9 cells infected with the recombinant baculovirus showed CPE at 24 hpi, whereas mock-infected cells exhibited no CPE as expected (Figures 2A,B). Furthermore, IFA analysis revealed a pronounced green fluorescence signal for infected Sf9 cells, whereas mock-infected cells showed no signal (Figures 2C,D). The RVFV Gn head-PA gene (1776bp) was detected in the P3 and P4 recombinant baculovirus by PCR and sequenced (Figure 2E). Last, Western blot analysis of both the supernatant and cell lysate from Sf9 cells infected with recombinant baculovirus revealed a 70 kDa band corresponding to the RVFV Gn head-PA fusion protein confirming its expression and secretion into the supernatant (Figure 2F).

### Analysis of Gram-Positive Enhancer Matrix Particles Loaded With Rift Valley Fever Virus Gn Head-Protein Anchor Fusion Protein

**FIGURE 3:**
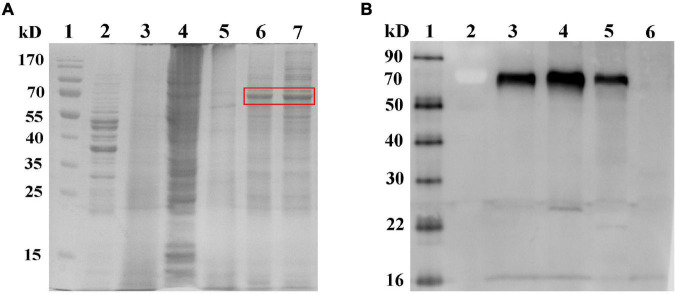
Identification of binding and dissociating of the RVFV Gn head-PA fusion protein to GEM particles. SDS-PAGE analyses of binding between RVFV Gn head-PA fusion protein and GEM particles **(A)**, lane 1 is protein marker, lane 2 is *L. lactis* MG1363, lane 3 is GEM particles, lane 4 is Sf9 cells, lane 5 is control baculovirus, lane 6 is RVFV-BLPs, and lane 7 is the supernatant containing RVFV Gn head-PA fusion protein. Western blot analyses of binding and dissociating between RVFV Gn head-PA fusion protein and GEM particles **(B)**, lanes 1 and 2 are protein marker, lane 3 is the supernatant after dissociated by using 2% Mercaptoethanol, lane 4 is RVFV-BLPs, lane 5 is the supernatant containing RVFV Gn head-PA fusion protein, and lane 6 is GEM particles.

RVFV-BLPs were subjected to both SDS-PAGE (Figure 3A) and Western blot (Figure 3B) analysis to confirm whether the RVFV Gn head-PA fusion protein was capable of binding to the GEM particles *via* PA. From the SDS-PAGE it can be observed that *L. lactis* MG1363 showed itself proteins (Figure 3A, lane 2) and GEM particles contained no proteins, confirming that treatment of *L. lactis* MG1363 with 10% TCA resulted in complete protein degradation (Figure 3A, lane 3). The RVFV-BLPs (Figure 3A, lane 6) contained fewer protein impurities by comparison with supernatant alone containing RVFV Gn head-PA fusion protein (Figure 3A, lane 7). In addition, Western blot analysis confirmed that the GEM particles alone contained no detectable RVFV Gn head-PA fusion protein as expected; the RVFV-BLPs confirmed the presence of the RVFV Gn head-PA fusion protein and were present in higher amounts when compared with supernatant alone containing RVFV Gn head-PA fusion protein (Figure 3B). Last, Western blot analysis of RVFV-BLPs treated with 2-mercaptoethanol revealed that the RVFV Gn head-PA fusion protein could be dissociated from the GEM particles following treatment with a reducing agent (Figure 3B). Together these data demonstrate that GEM particles can be used to enrich and purify the RVFV Gn head-PA fusion protein, and the non-covalent bond is very stable.

**FIGURE 4:**
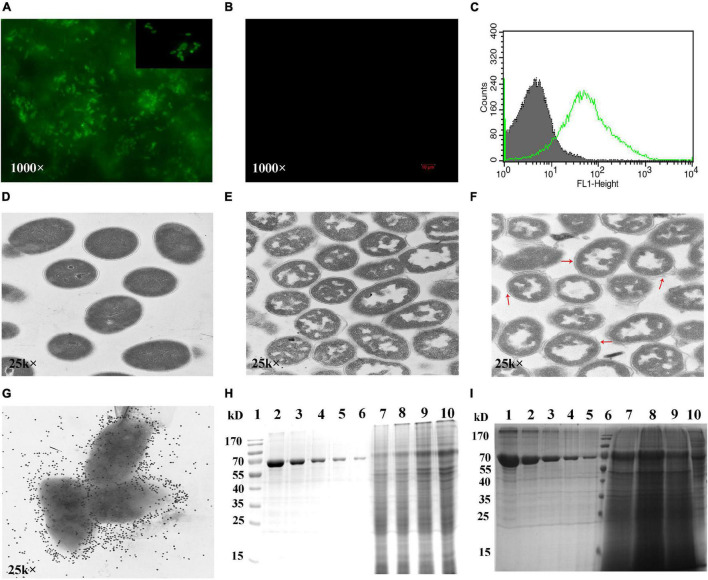
Detection of fusion protein displayed on the surface of GEM particles. IFA experiment detection of RVFV-BLPs labeled by goat antimouse FITC **(A)** and no fluorescence found in GEM particles **(B)**. FACS histograms of RVFV-BLPs (green) and GEM particles (gray) **(C)**. TEM and immunogold labeling images of RVFV-BLPs, *L. lactis* MG1363 **(D)**, GEM particles **(E)** and RVFV-BLPs **(F)**. Fusion protein on the surface of GEM particles detected by immunogold labeling **(G)**. The maximum binding capacity of the fusion protein displayed on GEM particles was determined by SDS-PAGE **(H,I)**. In **(H)**, lane 1 is protein marker, lanes 2–6 are BSA, 0.5–0.03125 mg/ml. 1U GEM particles mixed with 1 (lane 7), 2 (lane 8), 3 (lane 9), and 4 mL (lane 10). In **(I)**, lanes 1–5 are BSA, 0.5–0.03125 mg/ml. Lane 6 is protein marker, 1U GEM particles mixed with 6 (lane 7), 8 (lane 8), 10 (lane 9), and lane 10 is the supernatant containing RVFV Gn head-PA fusion protein.

Next, fluorescence microscopy, flow cytometry, TEM, and immuno-electron microscopy were performed to verify that the RVFV Gn head-PA fusion protein was displayed on the surface of the GEM particles. Immunofluorescence microscopy revealed that only the RVFV-BLPs showed fluorescence (Figure 4A), whereas the GEM particles alone did not (Figure 4B). Flow cytometry results showed that fluorescence was significantly increased in RVFV-BLPs compared with GEM particles (Figure 4C). Together, these results confirm that the RVFV Gn head-PA fusion protein was properly and efficiently displayed on the GEM particles. TEM was used to further evaluate differences in the morphology among untreated *L. lactis* MG1363 and GEM particles. Untreated *L. lactis* MG1363 had a uniform internal texture, and the bacterial cell wall was clearly visible (Figure 4D). In contrast, the GEM particles (*L. lactis* MG1363 treated with TCA) had structurally intact peptidoglycan with a morphology resembling a smooth outer sphere with a hollow interior (Figure 4E). Furthermore, the intracellular protein and DNA contents of the GEM particles appeared to be partially released or degraded as expected (Bosma et al., 2006). TEM analysis of the RVFV-BLPs revealed the RVFV Gn head-PA fusion protein adhered to the surface of the GEM particles like cotton wool (Figure 4F, red arrow). Last, immuno-electron microscopy revealed that the surface-exposed RVFV Gn head-PA fusion protein was evenly distributed on the GEM particles (Figure 4G).

### Maximum Binding Capacity of Rift Valley Fever Virus Gn Head-Protein Anchor Fusion Protein on Gram-Positive Enhancer Matrix Particles

The maximum binding capacity of the RVFV Gn head-PA fusion protein displayed on the GEM particles was evaluated by resuspending 1U of GEM particles in either 1, 2, 3, 4, 6, 8, or 10 mL of supernatant containing RVFV Gn head-PA fusion protein and subsequently subjected to SDS-PAGE. The result demonstrates that with the increase of supernatant volume binding with 1U GEM particles, the relative quality of binding increased (Figure 4H). Due to the similarity of the combination with 6, 8, and 10 mL, it was already saturated (Figure 4I, lane 7–9). Furthermore, we estimated that 1U GEM particles could bind 100 μg RVFV Gn head-PA fusion protein by Quantity One image analysis software.

### Detection and Measurement of Serum Antibody Response in Mice

**FIGURE 5:**
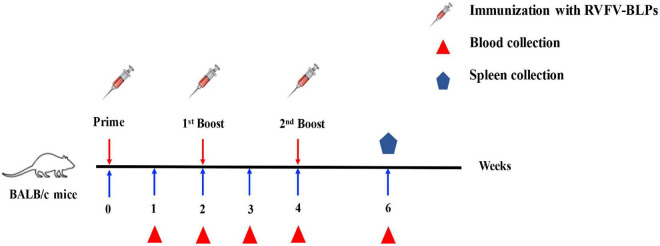
Immunization schedule. BALB/c mice were immunized intramuscularly with different doses of RVFV-BLPs and boosted two times with the same amount on days 14 and 28.

**FIGURE 6:**
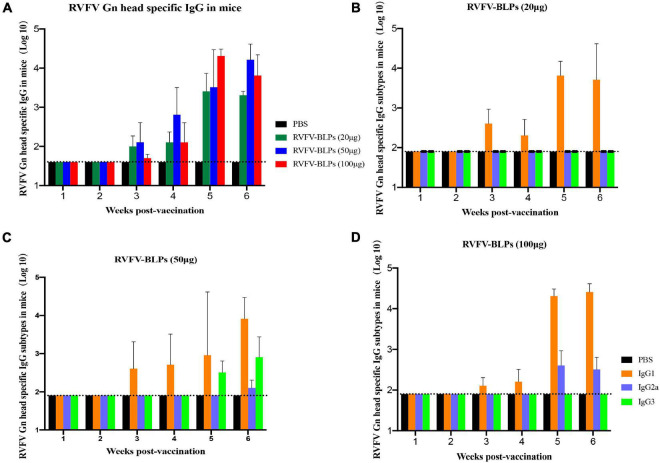
Detection of antibody levels in immunized mice sera. Sera collection from mice immunized with different amounts of RVFV-BLPs. RVFV Gn head-specific IgG (*p* > 0.05), IgG1, IgG2a, and IgG3 levels in the serum were assessed by indirect ELISA; data are shown as the means ± SD. RVFV Gn head-specific IgG levels in mice immunized with different amounts of RVFV-BLPs **(A)**, and the levels of IgG subtypes in RVFV-BLPs (20 μg) **(B)**, RVFV-BLPs (50 μg) **(C)** and RVFV-BLPs (100 μg) **(D)**. Dotted line is cutoff values, calculated as OD450 of control × 2.1. Data are shown as the mean ± SEM and were analyzed by an unpaired *t*-test.

BALB/c mice were immunized intramuscularly in groups of 10 with three different doses of RVFV-BLPs, namely, 20, 50, and 100 μg with control animals receiving PBS alone. Animals in each group received a prime immunization, followed by two repeated immunizations at 2 and 4 weeks post prime immunization and sera subsequently collected every week until 4 weeks after the second repeated immunization (Figure 5). For all groups, no RVFV Gn head specific IgG nor any of the IgG subtypes (IgG1, IgG2a, or IgG3) were detectable before 3 weeks post-primary immunization (Figure 6).

RVFV Gn head-specific IgG antibody titers were detectable for all groups starting at 3 weeks post-primary vaccination and peaked at 5 weeks post-primary vaccination, after which titers began to decrease with this decrease being most pronounced among the RVFV-BLPs (20 μg) group (Figure 6A). Interestingly, the comparison in IgG responses among the three different vaccine dose groups, revealed no positive correlation between antigen dose and the immunogenicity of the RVFV-BLPs, where the 50 μg dosage demonstrated the greatest immunogenicity, yielding the highest RVFV Gn head-specific IgG titers when compared with the 20 and 100 μg RVFV-BLP groups (Figure 6A). While the RVFV-BLPs (100 μg) group received the greatest amount of antigen, the appearance of RVFV Gn head-specific IgG antibodies was delayed compared with the 20- and 50 μg groups, only reaching comparable titers 4 weeks post-primary vaccination (Figure 6A).

To better characterize the antibody responses mounted against the RVFV Gn head protein, the levels of RVFV Gn head-specific IgG subtypes (IgG1, IgG2a, and IgG3) elicited following immunization with three different doses of RVFV-BLP were assessed by ELISA. The ratio of IgG2a/IgG1 is an indicator of Th1/Th2 deviation markers (type 1 cellular immune response or type 2 humoral immune response) with ratios > 1 being indicative of a Th1 response and ratios < 1 being indicative of a Th2 response. For all vaccine groups the IgG2a/IgG1 ratio was less than 1, suggesting that the RVFV-BLPs elicited a predominantly Th2 humoral immune response in BALB/c mice.

In all groups, RVFV Gn head-specific IgG1 subtype antibodies were detectable across all groups starting at 3 weeks post-primary vaccination (Figures 6B–D). In contrast, subtype IgG2a was only detectable in the 50 and 100 μg groups starting at 6 and 5 weeks post-primary immunization, respectively (Figures 6C,D) with no IgG2a being detected in the 20 μg group at any time point (Figure 6B). Surprisingly, RVFV Gn head-specific subtype IgG3 was only present in animals vaccinated with 50 μg RVFV-BLPs and was detectable starting at 5 weeks post-primary vaccination (Figure 6C). For all vaccinated animals, IgG1 was the dominant subtype induced by the RVFV-BLPs regardless of dosage.

### Rift Valley Fever Virus Neutralizing Activity

**FIGURE 7:**
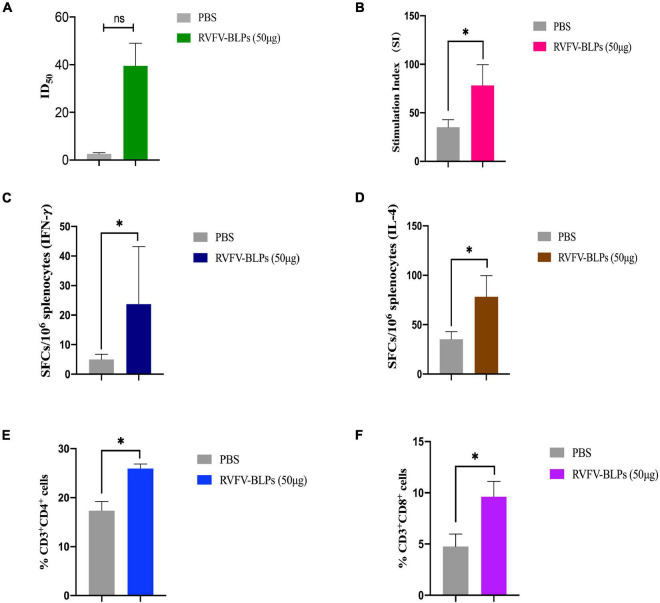
Humoral and cellular responses detection of mice immunized with RVFV-BLPs (50 μg). The neutralizing RVFV-pseudovirus activity of mice immunized with RVFV-BLPs (50 μg) of 2 weeks after the third immunization were represented by 50% inhibition dilution (ID_50_) **(A)**. Antigen-specific T cell proliferation, the stimulation index of the splenocytes was detected using a CCK assay by stimulating the cells with purified RVFV Gn head protein **(B)**. The RVFV Gn head-specific IFN-γ and IL-4 activity of splenocytes were evaluated using commercial ELISpot kits. SFC secreting IFN-γ **(C)** and IL-4 **(D)** were enumerated in an automated ELISpot reader. T cell proliferation was analyzed by using the following gating strategy: live*rightarrow*single cells*rightarrow*CD3^+^ → CD4^+^/CD8^+^ **(E,F)**. Data are shown as the mean ± SEM and were analyzed by an unpaired *t*-test (*p < 0.05).

Sera collected 6 weeks post-primary immunization from the RVFV-BLPs (50 μg) group was further analyzed to determine whether the anti-RVFV Gn head protein antibodies induced following vaccination exhibited neutralizing activity. The results demonstrate that immunized mice serum collected from 2 weeks after the third immunization could inhibit 50% RVFV pseudovirus infection (Figure 7A).

### Rift Valley Fever Virus-Bacterium-Like Particle Vaccines Induced T Cell Proliferative Responses

Splenocytes from mice immunized with RVFV-BLPs (50 μg) were harvested 6 weeks post-primary vaccination to evaluate whether immunization with RVFV-BLPs could induce antigen-specific T cell proliferation in the spleen. Splenocytes harvested from immunized mice were found to proliferate more efficiently than those from the PBS group (*p < 0.05) following stimulation with purified RVFV Gn head protein *in vitro* (Figure 7B).

### Rift Valley Fever Virus-Bacterium-Like Particle Vaccines Induced Antigen-Specific IFN-γ and IL-4 Secretion

The results demonstrate that RVFV-BLPs are capable of inducing the production of RVFV Gn head-specific antibodies and effectively stimulate splenocyte proliferative responses in immunized mice. As such, we set out to investigate whether RVFV-BLPs could promote cytokine secretion by evaluating through ELISpot assays IFN-γ and IL-4 responses in proliferating splenic T cells from immunized mice. From the results, it can be observed that the levels of IFN-γ and IL-4 in splenocytes harvested from the RVFV-BLPs (50 μg) group were significantly higher than those in the PBS control group (*p < 0.05) (Figures 7C,D), indicating that RVFV-BLPs could promote cytokine secretion.

### Rift Valley Fever Virus-Bacterium-Like Particle Vaccines Induced Antigen-Specific Cellular Immune Response

To evaluate the cellular immune response induced by RVFV-BLPs, splenocytes from the RVFV-BLPs (50 μg) group were harvested 6 weeks post-primary vaccination and subjected to flow cytometry to characterize T cell subset populations in splenocytes (Figures 7E,F). Analysis by flow cytometry revealed that the number of CD3^+^CD4^+^ (Figure 7E) and CD3^+^CD8^+^ (Figure 7F) T cells in splenocytes harvested from mice immunized with 50 μg RVFV-BLPs were significantly elevated (*p < 0.05) when compared with the PBS control group.

## Discussion

While several types of RVFV vaccines (Barteling and Woortmeyer, 1984; Minke et al., 2004; Bhardwaj et al., 2010; Kortekaas, 2014; Chamchod et al., 2015; Said et al., 2017) have been reported in recent years, there still remains no fully licensed vaccines for use in non-endemic countries for either humans or veterinary animals. In contrast, a limited number of vaccines are available for veterinary use in endemic countries; however, there is no clear policy or practice of routine livestock vaccinations as a preventive strategy against potential RVF disease outbreaks. In this study, we describe a novel RVFV-BLPs vaccine platform, consisting of *L. lactis* MG1363–derived GEM particles displaying on their surface the RVFV Gn head-PA fusion protein *via* a transmembrane protein anchor. The display of large antigens on bacterial surfaces can at times be problematic as they perturb the membrane topology (Lee et al., 2006), and as such, to overcome this, we used the head domain of RVFV Gn starting with the fifth ATG (426–1,427 nt), which is an ideal region recognized by neutralizing antibodies (Wu et al., 2017). Secretory expression is a major criterion for large-scale production of vaccines, and as such, an HBM signal was added upstream of the RVFV Gn head domain (Figure 1) to allow for secretion of the RVFV Gn head-PA fusion protein into the supernatant following infection of Sf9 cells with the constructed recombinant baculovirus. Sf9 cells infected with the constructed recombinant baculovirus displayed CPE within 24 h post infection (Figures 2A,B), and not only was the fusion protein expressed (Figure 2D), but it was also secreted into the supernatant (Figure 2F).

The GEM particles used to display the RVFV Gn head-PA fusion protein were prepared through treatment of *L. lactis* MG1363 with 10% TCA. Following treatment, the GEM particles appeared as peptidoglycan spheres with all proteins and intracellular DNA degraded as observed by TEM (Figure 4E). In contrast, TEM of untreated *L. lactis* MG1363 revealed an intact bacterial cell wall (Figure 4D). Both SDS-PAGE and Western blot analysis of the RVFV-BLPs confirmed that the RVFV Gn head-PA fusion protein was bound to the GEM particles (Figures 3, 4) and displayed on the surface of the GEM particles *via* PA with an appearance similar to cotton filaments as observed by TEM (Figures 4A,F,G). Last, the maximum amount of RVFV Gn head-PA fusion protein capable of binding to 1 U (2.5 × 10^9^ particles) of GEM particles was empirically determined to be 100 μg (Figures 4H,I).

To evaluate the immunogenicity of the RVFV-BLPs, BALB/c mice were immunized intramuscularly with three different doses, namely, 20, 50, and 100 μg of RVFV-BLPs. Mice were given a prime immunization followed by two repeated immunizations at 2 and 4 weeks post prime immunization and sera harvested weekly until 4 weeks after the second repeated (third immunization) (Figure 5). For all groups, RVFV Gn head-specific IgG antibodies were detectable 1 week after the second immunization; however, the 50 μg group had the highest titers compared with the 20 and 100 μg groups (Figure 6A). The specific IgG in the 50 μg group showed higher than the 100 μg group in early immunization and faded slowly. Further evaluation of the IgG subtypes (IgG1, IgG2a, and IgG3) among the three vaccine doses revealed that the level of the IgG subtypes had no linear relationship with the level of IgG, and the RVFV-BLPs elicited a predominantly Th2 humoral immune response in BALB/c mice.

The comprehensive analysis of the RVFV Gn head-specific IgG suggests that the RVFV-BLPs were capable of inducing a humoral immune response. With these findings, we selected the RVFV-BLPs (50 μg) group for further investigation to test for the presence of neutralizing antibodies as well as characterize the cellular immune response induced by the RVFV-BLPs. The immunized mice serum harvested 2 weeks after the second repeated immunization was evaluated for neutralizing activity by using a RVFV pseudovirus and revealed that mice immunized with RVFV-BLPs could produce a neutralizing RVFV pseudovirus antibody (Figure 7A). Together, these data reveal that BALB/c mice immunized with RVFV-BLPs mount a productive humoral immune response. For cellular immune response, the results show that RVFV-BLPs (50 μg) can induce antigen-specific T cell proliferation in the spleen (Figure 7B) and also effectively stimulate splenocytes to secrete IFN-γ (Figure 7C) and IL-4 (Figure 7D). IFN-γ is a Th1-type cytokine involved in the cellular immune response, and IL-4 is a Th2-type cytokine associated with humoral immune responses, which indicates that RVFV-BLPs (50 μg) could effectively stimulate the production of both Th1 and Th2 cytokines in mice, thereby enhancing cellular and humoral immune responses.

Last, we further characterize the cellular immune response mounted following immunization with 50 μg RVFV-BLPs and find that vaccination with RVFV-BLPs significantly increased numbers of CD4^+^ (Figure 7E) and CD8^+^ (Figure 7F) T cells compared with the PBS control group.

In short, the described RVFV-BLPs were found to induce both humoral and cellular immune responses in immunized BALB/c mice and, thus, represent a novel and promising vaccine candidate for the prevention of RVF infection in both humans and veterinary animals. Furthermore, the low cost and scalable production of the RFVF BLPs make them appealing for immunization on a large scale. Last, an additional benefit of RVFV-BLPs is that they could enable differentiation between infected and non-infected vaccinated animals (DIVA). While these RVFV-BLPs represent a novel vaccine candidate, further investigation is required to determine whether they can confer protection *in vivo* following challenge with RVFV.

## Data Availability Statement

The original contributions presented in the study are included in the article/supplementary material, further inquiries can be directed to the corresponding author/s.

## Ethics Statement

The animal study was reviewed and approved by the Welfare and Ethics of Laboratory Animals of China (GB 14925-2001).

## Author Contributions

SZ and FY conceived and designed the experiments. SZ, FY, and EL performed the experiments. DL, HW, and YG analyzed the data. SZ wrote the manuscript. XX, YZ, and SY reviewed the manuscript. All authors read and approved the final manuscript.

## Conflict of Interest

The authors declare that the research was conducted in the absence of any commercial or financial relationships that could be construed as a potential conflict of interest.

## Publisher’s Note

All claims expressed in this article are solely those of the authors and do not necessarily represent those of their affiliated organizations, or those of the publisher, the editors and the reviewers. Any product that may be evaluated in this article, or claim that may be made by its manufacturer, is not guaranteed or endorsed by the publisher.
